# HBx and c-MYC Cooperate to Induce URI1 Expression in HBV-Related Hepatocellular Carcinoma

**DOI:** 10.3390/ijms20225714

**Published:** 2019-11-14

**Authors:** Hiroyuki Tsuchiya, Masataka Amisaki, Ai Takenaga, Soichiro Honjo, Yoshiyuki Fujiwara, Goshi Shiota

**Affiliations:** 1 Division of Molecular and Genetic Medicine, Graduate School of Medicine, Tottori University, 86 Nishi-cho, Yonago 683-8503, Japan; 2 Division of Surgical Oncology, Department of Surgery, Faculty of Medicine, Tottori University, 86 Nishi-cho, Yonago 683-8503, Japan

**Keywords:** URI1, HBx, c-MYC, HBV-related hepatocellular carcinoma, E-box

## Abstract

Unconventional prefoldin RNA polymerase II subunit 5 interactor (URI1) has emerged as an oncogenic driver in hepatocellular carcinoma (HCC). Although the hepatitis B virus (HBV) represents the most common etiology of HCC worldwide, it is unknown whether URI1 plays a role in HBV-related HCC (HCC-B). In the present study, we investigated URI1 expression and its underlying mechanism in HCC-B tissues and cell lines. URI1 gene-promoter activity was determined by a luciferase assay. Human HCC-B samples were used for a chromatin immunoprecipitation assay. We found that c-MYC induced URI1 expression and activated the *URI1* promoter through the E-box in the promoter region while the HBx protein significantly enhanced it. The positivity of URI1 expression was significantly higher in HCC-B tumor tissues than in non-HBV-related HCC tumor tissues, suggesting that a specific mechanism underlies URI1 expression in HCC-B. In tumor tissues from HCC-B patients, a significantly higher level of c-MYC was recruited to the E-box than in non-tumor tissues. These results suggest that HBx and c-MYC are involved in URI1 expression in HCC-B. URI1 expression may play important roles in the development and progression of HCC-B because HBx and c-MYC are well-known oncogenic factors in the virus and host, respectively.

## 1. Introduction

Hepatitis B virus (HBV) represents the most common etiology of hepatocellular carcinoma (HCC) worldwide [1]. In Japan, the number of patients who die of HBV-related hepatocellular carcinoma (HCC-B) has not changed over the last decade, even though several anti-HBV drugs that prevent the onset of HCC-B are available [2]. The number of HCC-B patients is gradually and consistently increasing at the global level, and this increase is more prominent in countries with a high socio-demographic index score [1]. It has been shown that antiviral therapy with entecavir or tenofovir reduces HCC development in chronic hepatitis B patients. However, the yearly HCC incident was still non-negligible, even after five-years of treatment [3]. These facts emphasize the importance of developing a drug with a novel mechanism of action.

The HB protein encoded by the *HBV X* (*HBx*) gene is a potent oncogenic factor that interacts with numerous host transcription factors [4,5], of which c-MYC is one of the best characterized. HBx increases cellular c-MYC stability by inhibiting the ubiquitination of c-MYC, and promotes c-MYC-induced oncogenesis [6,7]. The *HBx* gene, driven by an HBV-native promoter, consistently potentiates c-MYC-induced hepatocarcinogenesis in mice [8]. Clinically, HBV integration near the *c-MYC* gene was found at a significantly higher frequency in early-onset HCC-B than in late-onset HCC-B [9]. These findings suggest that c-MYC and its target genes may facilitate the development of novel therapeutics to treat HCC-B.

Unconventional prefoldin RNA polymerase II subunit 5 (RPB5) interactor (*URI1*) has emerged as an oncogene in HCC that induces DNA damage in hepatocytes [10,11]. Its upregulation decreases expression of enzymes required for NAD + biosynthesis, and thereby suppresses NAD + -dependent DNA repair enzymes [10]. This URI1-induced DNA damage not only directly induces liver tumorigenesis but also enhances Th17 cell-mediated inflammation, which leads to non-alcoholic steatohepatitis and HCC [10,11]. Moreover, URI1 reportedly promotes metastatic invasion of HCC [12]. These observations suggest that URI1 plays critical roles in the development and progression of HCC.

The expression of URI1 in the liver is regulated by inflammation, nutrition, and hepatitis virus infection [10,11]. Tummala et al. demonstrated that HBx enhances URI1 expression, and that HCC in human URI1 transgenic mice has a similar overlap in transcriptional profiles with HCC-B in human patients [10], suggesting that HBV employs URI1 to develop and progress HCC. However, another group reported that HBx does not modulate the expression of URI1, but these proteins interact with each other to promote the growth of HCC [13]. As the precise mechanism of URI1 expression in HCC-B remains unclear, we investigated the mechanism underlying how HBx induces URI1 expression.

## 2. Results

### 2.1. URI1 Promoter Activation by HBx and c-MYC

**Figure 1:**
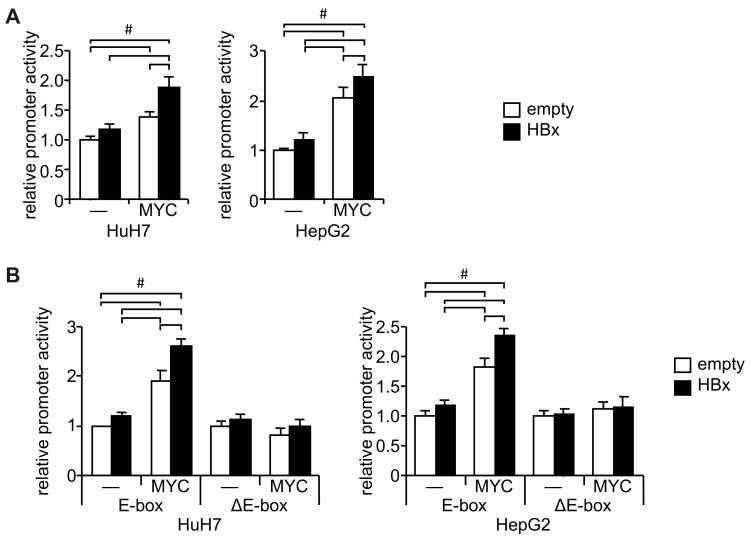
The unconventional prefoldin RNA polymerase II subunit 5 interactor (URI1) promoter activation by HBx and c-MYC through E-box. (**A**) A reporter plasmid under the control of the *URI1* promoter (−304/+67; Supplementary Figure S1A) was co-transfected into HuH7 (**left**) and HepG2 (**right**) cells with HBx- or c-MYC-expressing plasmids. (**B**) Reporter plasmids for the *URI1* promoter with wild-type or mutant E-boxes (E-box and ΔE-box, respectively; Supplementary Figure S1) were co-transfected into HuH7 (left) and HepG2 (right) cells with HBx- or c-MYC-expressing plasmids. Luciferase assays were performed 2 days post-transfection. pCMV-Flag, and pGL4.74[hRluc/TK] were used as empty and transfection controls, respectively. Data are shown as mean ±SD (*n* = 3–4). #; *p* < 0.05 was determined by Tukey’s test.

A reporter assay showed that the *URI1* promoter was significantly activated by HBx even when it was shortened to −304 bp (Supplementary Figure S1A,B). The ENCODE project [14] revealed that this region (GRCh37/hg19: chr19: 30,432,842–30,433,213) includes the biding site of c-MYC (Supplementary Figure S2A) and a CACGCG non-canonical E-box, reportedly one of the major c-MYC-binding sites [15], which was identified by the JASPAR database in the −109 to −104 region [16] (Supplementary Figure S2B). Our examination of the effect of c-MYC on the *URI1* promoter showed that c-MYC increased the *URI1* promoter activity, and HBx significantly enhanced the effect in both HuH7 and HepG2 cells (Figure 1A). Although weak promoter activation by HBx alone was observed (Figure 1A), in contrast to the results shown in Supplementary Figure S1B, this may have been because the amount of plasmid DNA required for co-transfection was reduced to half that required for single transfection. While the promoter region encompassing −183 to +67 responded to HBx and c-MYC co-transfection, this response was no longer observed with the promoter region from −99 to + 67 (Supplementary Figure S1A,C). Removal of the putative E-box abrogated the response to HBx and c-MYC (Figure 1B, Supplementary Figure S2B). These results suggest that HBx and c-MYC increased the activity of the *URI1* promoter through the non-canonical E-box.

### 2.2. Induction of URI1 Expression by HBx and c-MYC

**Figure 2:**
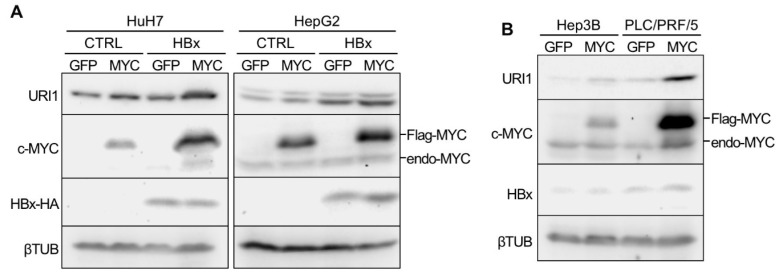
URI1 protein expression induced by HBx and c-MYC. (**A**) Protein expression of URI1, c-MYC, HA-tagged HBx (HBx-HA) and βTUB in control (CTRL) or HBx-overexpressing HuH7 and HepG2 cells at 2 days post-transduction of adenovirus vectors expressing c-MYC (MYC) or AcGFP (GFP). Flag-MYC, FLAG-tagged c-MYC and endo-MYC, endogenous c-MYC. (**B**) Protein expression of URI1, c-MYC, HBx, and βTUB in Hep3B and PLC/PRF/5 cells at 2 days post-transduction of AdMYC (MYC) or AdGFP (GFP).

On its own, c-MYC markedly induced the expression of *URI1* mRNA in HuH7 cells (Supplementary Figure S3A). In contrast, in HepG2 cells, marked induction of *URI1* mRNA was observed by HBx rather than by c-MYC (Supplementary Figure S3A). However, the co-overexpression of c-MYC and HBx significantly increased *URI1* mRNA expression, compared with either alone, in both cell lines (Supplementary Figure S3A). URI1 protein expression was consistently increased by HBx and c-MYC (Figure 2A). As previously reported [6,7], exogenous c-MYC protein (Flag-MYC) was stabilized more in the HBx-expressing cells than in control cells (Figure 2A). HBx alone did not show a marked effect on both mRNA and protein expressions of URI1 in HuH7 cells (Figure 2A, Supplementary Figure S3A). This might be explained by the relatively low expression of the endogenous c-MYC protein in HuH7 cells, in contrast to HepG2 cells (Figure 2A). The Hep3B and PLC/PRF/5 cell lines, which are derived from HCC-B, also showed increased URI1 mRNA and protein expression by c-MYC overexpression (Figure 2B, Supplementary Figure S3B). These results suggest that HBx induces URI1 expression in HCC-B via the host oncoprotein c-MYC.

### 2.3. URI1 Expression in HCC-B Tissues

**Figure 3:**
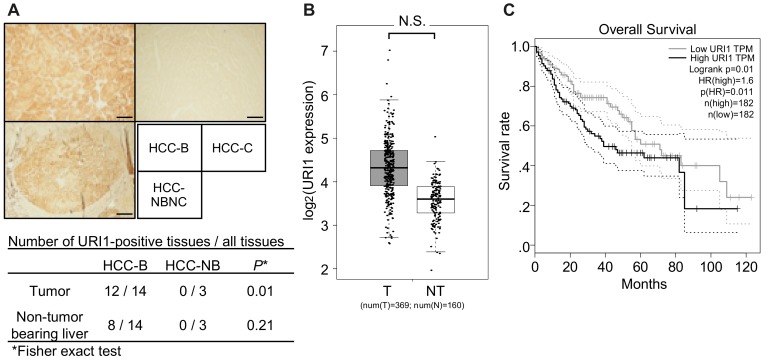
URI1 expression in HCC-B tumor tissues. (**A**) Representative images of URI1 immunohistochemistry of URI1-positive HCC-B (**upper left**), URI1-positive non-HBV-related HCC (**lower left**), and URI1-negative HCC-C (**upper right**). Scale bars, 500 µm. Summary of URI1-positive tissues is shown below the images. The *p*-values were calculated by Fisher’s exact test. (**B**) *URI1* mRNA expression in HCC (T; filled bar) and normal liver (NT; open bar) tissues. N.S., not significant (one-way ANOVA). (**C**) Kaplan–Meier analysis of the overall survival of patients with high or low URI1 expression levels in HCC tissues. The RNA-seq data were retrieved from the TCGA database.

Immunohistochemistry analysis of URI1 found that the positivity of URI1 expression was significantly higher in HCC-B tumor tissues than in non-HBV-related HCC tumor tissues, while no significant difference was observed in surrounding non-tumor-bearing liver tissues (Figure 3A).

RNA-seq data retrieved from The Cancer Genome Atlas (TCGA) and genotype-tissue expression (GTEx) showed a tendency for increased expression levels of URI1 in HCC tissues, compared with normal livers, although the difference was not statistically significant (Figure 3B). When HCC patients, which include those with various risk factors, such as alcohol consumption (31.9%), HBV (27.8%), HCV (15.8%), NAFLD (3.8%), and others [17], were stratified by mean expression of URI1, a lower overall survival rate was seen compared with low URI1 expression (Figure 3C). These observations were similar to those reported previously [10].

### 2.4. Involvement of c-MYC in URI1 Expression in HCC-B

**Figure 4:**
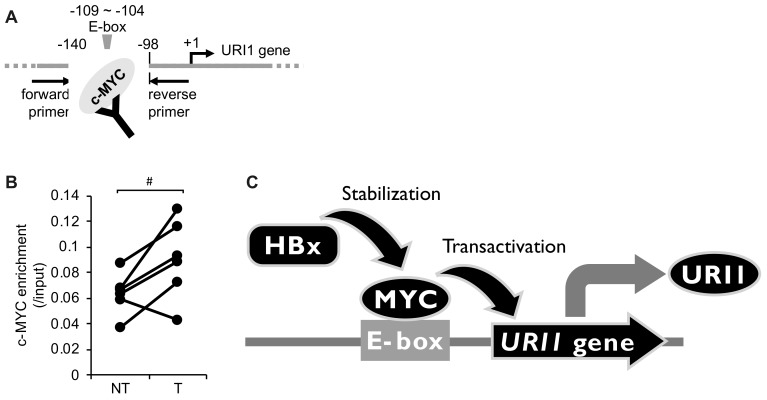
Recruitment of c-MYC to the URI1 gene promoter in HCC-B tumor tissues. (**A**,**B**) Following chromatin immunoprecipitation (ChIP) with anti-c-MYC antibody, qPCR was performed with primers indicated in the figure. #; *p* < 0.05 was determined by paired Student’s t-test. NT, non-tumor tissues and T, tumor tissues from HCC-B patients. (**C**) Model describing HBx-induced URI1 expression through c-MYC in HCC-B. c-MYC induces URI1 expression through a non-canonical E-box in the URI1 promoter. HBx potentiates this action by enhancing the intracellular stability of c-MYC.

We performed a chromatin immunoprecipitation (ChIP) assay in paired tumor and non-tumor liver tissues from HCC-B patients to investigate whether c-MYC plays a role in URI1 expression in HCC-B. It was shown that c-MYC recruitment to the E-box in the URI1 promoter region was significantly increased in HCC-B tumor tissues, compared with paired non-tumor liver tissues (Figure 4A,B), suggesting that c-MYC is involved in the transcription of the *URI1* gene in HCC-B.

## 3. Discussion

In the present study, we demonstrated that HCC-B tumor tissues frequently express URI1 proteins, and that HBx activates the promoter of the *URI1* gene. HBx does not harbor a DNA-binding domain, it rather indirectly modulates a number of target genes by affecting epigenetic machineries or signal transduction factors [18]. To identify the factor mediating HBx-induced URI1 expression, a search was conducted for putative transcription factor binding sites in the *URI1* promoter. Finally, we demonstrated that c-MYC is involved in the mechanism underlying HBx-induced URI1 expression, and that the E-box in the *URI1* promoter is a biding site for c-MYC in HCC-B (Figure 4C). Although current clinical guidelines prioritize the treatment of HCC, it is also suggested that patients with HCC-B should undergo antivirus therapy prior to HCC treatment to reduce the risks of further liver injury, HBV reactivation, and HCC recurrence [19]. Therefore, the appropriate management of chronic HBV infection in HCC is attracting attention. In this context, URI1 would be an ideal target for the treatment of HCC-B because it is involved in both hepatitis and HCC [10,11].

The canonical E-box sequence consists of CACGTG, and is employed as a binding site for basic helix-loop-helix transcription factors including c-MYC [20]. However, a genome-wide search for c-MYC binding sites in HeLa cells and human fibroblasts identified 1469 c-MYC target genes, and the major consensus sequences found in those sites were CACGCG as well as CACGTG [15]. Our study demonstrated that the non-canonical CACGCG E-box functions as a c-MYC binding site in HCC-B, and that c-MYC binds to the CACGCG motif in the *NAMPT* gene promoter in a breast cancer cell line [21]. These findings suggest that transcriptional regulation via the CACGCG motif by c-MYC may be a prevalent mechanism in tumors. However, because another MYC family member, N-MYC, also reportedly binds to the CACGCG motif in the *WDR5* gene promoter [22], we cannot rule out the involvement of N-MYC, which plays a critical role in liver cancer stem cells [23].

URI1 is an RPB5 interactor that inhibits a part of RNA polymerase II-directed transcription [24]. HBx also binds to RPB5, but not to URI1, and forms a trimeric complex with the general transcriptional factor IIB, leading to the transactivation of the HBV promoter [24,25]. Interestingly, it has been demonstrated that URI1 and HBx compete with each other for transcription [24]. These results suggest that URI1 may suppress HBV replication in the nucleus. However, we observed cytosolic localization of URI1 in HepG2 control and HBx cells (A.T., personal communication), in agreement with a previous report that found that URI1 inhibits nuclear localization of nuclear receptors by sequestering them in the cytosol [10]. In HCC-B tumor tissues, immunostaining of URI1 was also detected mainly in the cytosol, but also in the nucleus. Studies are currently underway to clarify the function of URI1 in the development and progression of HCC-B.

URI1 is also reportedly involved in c-MYC regulation [26]. Under glucose deprivation, protein kinase A phosphorylates URI1, which then suppresses O-linked N-acetylglucosamine (GlcNAc) transferase (OGT). Phosphorylation of c-MYC at Thr57 induces its proteasomal degradation while O-GlcNAcylation at Thr57 increases the protein level of c-MYC [27,28]. Thus, URI1-induced OGT suppression depletes c-MYC protein [26]. Cancer cells require this regulatory mechanism because c-MYC overexpression induces apoptosis of cancer cells under glucose deprivation [29]. In this context, URI1 induced by c-MYC may function as a negative-feedback regulator that allows cancer cells to adapt to metabolic stress and survive under glucose deprivation. When glucose is abundant, dephosphorylated URI1 stabilizes c-MYC proteins by activating OGT, thereby promoting c-MYC-dependent tumorigenesis [26]. A recent paper reported that HBx also protects HCC cells from glucose deprivation-induced metabolic stress by facilitating fatty-acid oxidation [30]. These findings suggest that metabolic reprogramming may be one possible function of URI1 in HCC-B.

Sorafenib, a molecular targeted drug for HCC, was shown to be effective for patients with well-preserved liver function (Child-Pugh A) although poor outcomes were significantly associated with Child-Pugh B patients [31]. A natural substance, betulinic acid, sensitized pancreatic ductal adenocarcinoma cells to sorafenib accompanying the downregulation of c-MYC [32]. Thus, it is also worth it to investigate whether c-MYC-induced URI1 is involved in sorafenib resistance mechanisms in HCC-B patients with decompensated cirrhosis.

The WNT/β-catenin signaling pathway is an important driver for hepatocarcinogenesis [33]. However, the frequency of mutations in the *CTNNB1* gene is much lower in HCC-B than in non-HBV-related HCC [34]. This is consistent with the finding that HBx activates WNT/β-catenin signaling [35]. In addition, it was shown that URI1 was involved in the activation of WNT/β-catenin signaling by a long non-coding RNA in cardiac fibroblasts [36]. Therefore, it might be plausible that the activation of WNT/β-catenin signaling in HCC-B may depend on HBx and URI1.

It is suggested that estrogen has a suppressive effect on HCC because it is known that the prevalence of HCC is high in males, and low in premenopausal females [37]. It is also implied that there are sex differences in response to HBV infection [38]. It was demonstrated that URI1 inhibits NAD synthesis by suppressing the function of estrogen receptor in hepatocytes, leading to the development of HCC [10]. The cell lines used in the study were all derived from male HCC. Thus, it is intriguing to investigate the involvement of URI1 in the sex difference by using a female HCC-B cell line [39].

In conclusion, we revealed the HCC-B-specific mechanism that induces *URI1* gene expression by HBx through c-MYC. The precise pathogenic role of URI1 in HCC-B remains to be clarified. However, given that URI1 plays critical roles in HCC, as previously reported, and that HBx and c-MYC are well-known oncogenic factors, this transcriptional regulatory mechanism may be a potential therapeutic target for HCC-B. Because c-MYC may be involved in early-onset HCC-B [9], URI1-targeted therapy may be a more effective treatment for early-onset HCC-B compared with other HCCs.

## 4. Materials and Methods

### 4.1. Materials

Hep3B and PLC/PRF/5 cells lines were obtained from the Cell Resource Center for Biomedical Research, Institute of Development, Aging and Cancer at Tohoku University, and the Japanese Collection of Research Bioresources Cell Bank, respectively. HuH7, HepG2, pCMV-Flag (RDB05956), and pCMV-FlagMYC (RDB06671) were provided by the RIKEN BRC through the National Bio-Resource Project of the MEXT, Japan. pcDNA3-Hbxadr-Hatag was a gift from Xin Wang (# 24930; Addgene, Cambridge, MA, USA) [40]. pAcGFP1-C1 was purchased from Clontech (Palo Alto, CA, USA). pGL4.10[luc2], pGL4.74[hRluc/TK], and Dual-Luciferase Reporter Assay System were purchased from Promega (Madison, WI, USA). BP clonase, LR clonase, pDONR-221, and pAd/CMV/V5-DEST were purchased from Thermo Fisher Scientific (Cleveland, OH, USA). Antibodies were purchased as follows, anti-HA tag (16B12; BioLegend), anti-HBx (3F6-G10; Novus Biologicals, Centennial, CO, USA), anti-c-MYC (9E1; Santa Cruz Biotechnology, Santa Cruz, CA, USA), anti-β-tubulin (βTUB; G-8; Santa Cruz Biotechnology), anti-URI1 (SP215; Abcam, Cambridge, MA, USA), and horseradish peroxidase-conjugated anti-mouse immunoglobin G (IgG) and anti-rabbit IgG antibodies (Cell Signaling Technology, Danvers, MA, USA). Anti-c-MYC antibody (MC045)- and anti-mouse IgG-conjugated agarose beads were purchased from Nacalai (Kyoto, Japan) and Novus Biologicals (Centennial, CO, USA), respectively. Primers were purchased from Fasmac (Atsugi, Japan), and are summarized in Supplementary Table S1.

### 4.2. Cell Culture

The cells were maintained in Dulbecco’s Modified Eagle Medium (Nissui, Tokyo, Japan) supplemented with 4 mM of L-glutamine, and 10% fetal bovine serum in a humidified atmosphere at 37 °C and 5% CO_2_. The medium for culture of PLC/PRF/5 cells was additionally supplemented with 4.5 g/L glucose. HBx-overexpressing HuH7 and HepG2 cells and their control cells were established by stably transfecting pcDNA3-Hbxadr-Hatag and pCMV-Flag, respectively, followed by G418 selection. HBx expression was confirmed by quantitative polymerase chain reaction (qPCR; Supplementary Figure S3A).

### 4.3. Immunohistochemistry and ChIP Assay

Patients with HCC are summarized in Supplementary Table S2. Formalin-fixed paraffin-embedded tissue specimens were cut into 4 μm sections. After dewaxing in xylene, tissue sections were autoclaved for 10 min in a citrate buffer, pre-incubated in 0.3% H_2_O_2_, and blocked for 20 min using diluted normal blocking serum from a VECTASTAIN Elite ABC HRP kit (Vector Laboratories, Burlingame, CA, USA). Sections were incubated with 1:100 diluted anti-URI1 antibody as a primary antibody followed by an HRP-conjugated secondary antibody (Vector Laboratories). URI1 protein was visualized using ImmPACT DAB substrate (Vector Laboratories) with a reaction time of 3 min.

Paired HCC-B and non-tumor liver tissues from five patients (Supplementary Table S3) were used for the ChIP assay, which was performed as previously reported [41].

These studies were approved by the ethical committee of Tottori University (18A071).

### 4.4. Plasmid Construction

The promoter region of the human *URI1* gene was amplified with KOD-plus-NEO (TOYOBO, Otsu, Japan), and ligated into the luciferase plasmid. Mutagenesis reaction was performed by QuikChange Lightning Site-Directed Mutagenesis Kits (Agilent Technologies, Santa Clara, CA, USA) as previously reported [42]. Fragments of FLAG-tagged c-MYC and V5-tagged AcGFP were amplified by KOD-plus-NEO and transferred into the adenovirus plasmid using Gateway technology according to the manufacturer’s instruction.

### 4.5. Reporter Assay, Western Blotting, qPCR

Promoter activity and protein and mRNA expression levels were determined as previously reported [42]. Transfection was performed with Viofectin (Viogene, Taipei, Taiwan). Gene transduction with an adenovirus vector was performed at a multiplicity of infection of 50 per day following cell seeding. Protein and mRNA were recovered from the cells 48 hours post-transduction.

### 4.6. Analysis of TCGA Data Set

URI1 expression in TCGA and GTEx RNA-seq datasets of HCC and normal livers were analyzed using GEPIA 2 [43]. The difference in gene expression and overall survival was analyzed by one-way analysis of variance (ANOVA) and log-rank test, respectively. The data were accessed on June 27, 2019.

### 4.7. Statistical Analysis

Independent samples, of which numbers are over three, were analyzed, and all experimental values were expressed as mean ± standard deviation (SD). The differences between the two groups were assessed by Student’s *t*-test or Tukey’s test. A *p*-value < 0.05 was considered statistically significant.

## Supplementary Materials

Supplementary materials can be found at https://www.mdpi.com/1422-0067/20/22/5714/s1.

## Abbreviations

ChIP	chromatin immunoprecipitation
GTEx	genotype-tissue expression
HBV	hepatitis B virus
HCC	hepatocellular carcinoma
HCC-B	hepatitis B virus-related hepatocellular carcinoma
HCC-NB	non-hepatitis B virus-related hepatocellular carcinoma
qPCR	quantitative polymerase chain reaction
TCGA	The Cancer Genome Atlas
URI	unconventional prefoldin RPB5 interactor
