# Supplementary material for: HBx and c-MYC Cooperate to Induce URI1 Expression in HBV-Related Hepatocellular Carcinoma
Source: Int J Mol Sci. 2019 Nov 14;20(22):5714. doi: 10.3390/ijms20225714 (PMC6888623; doi:10.3390/ijms20225714)
Supplement: Supplementary file 1 [file ijms-20-05714-s001.zip › SupFiles/SupFig.pdf]

Supplementary Figures

HBx and c-MYC cooperate to induce URI1 expression in HBV-related hepatocellular carcinoma

Hiroyuki Tsuchiya [1,*], Masataka Amisaki [2], Ai Takenaga [1], Soichiro Honjo [2], Yoshiyuki Fujiwara [2] and Goshi Shiota [1]

[1] Division of Molecular and Genetic Medicine, Graduate School of Medicine, Tottori University, 86 Nishi-cho, Yonago, 683-8503, Japan.; tsuchiyah@tottori-u.ac.jp (H.T.); M18M9007B@edu.tottori-u.ac.jp (A.T.); gshiota@tottori-u.ac.jp (G.S.)
[2] Division of Surgical Oncology, Department of Surgery, Faculty of Medicine, Tottori University, 86 Nishi-cho, Yonago, 683-8503, Japan.; amisakim@gmail.com (M.A.); honjo@tottori-u.ac.jp (S.H.); y-fujiwara@tottori-u.ac.jp (Y.F.)
* Correspondence: tsuchiyah@tottori-u.ac.jp; Tel/Fax.: +81-859-38-6435

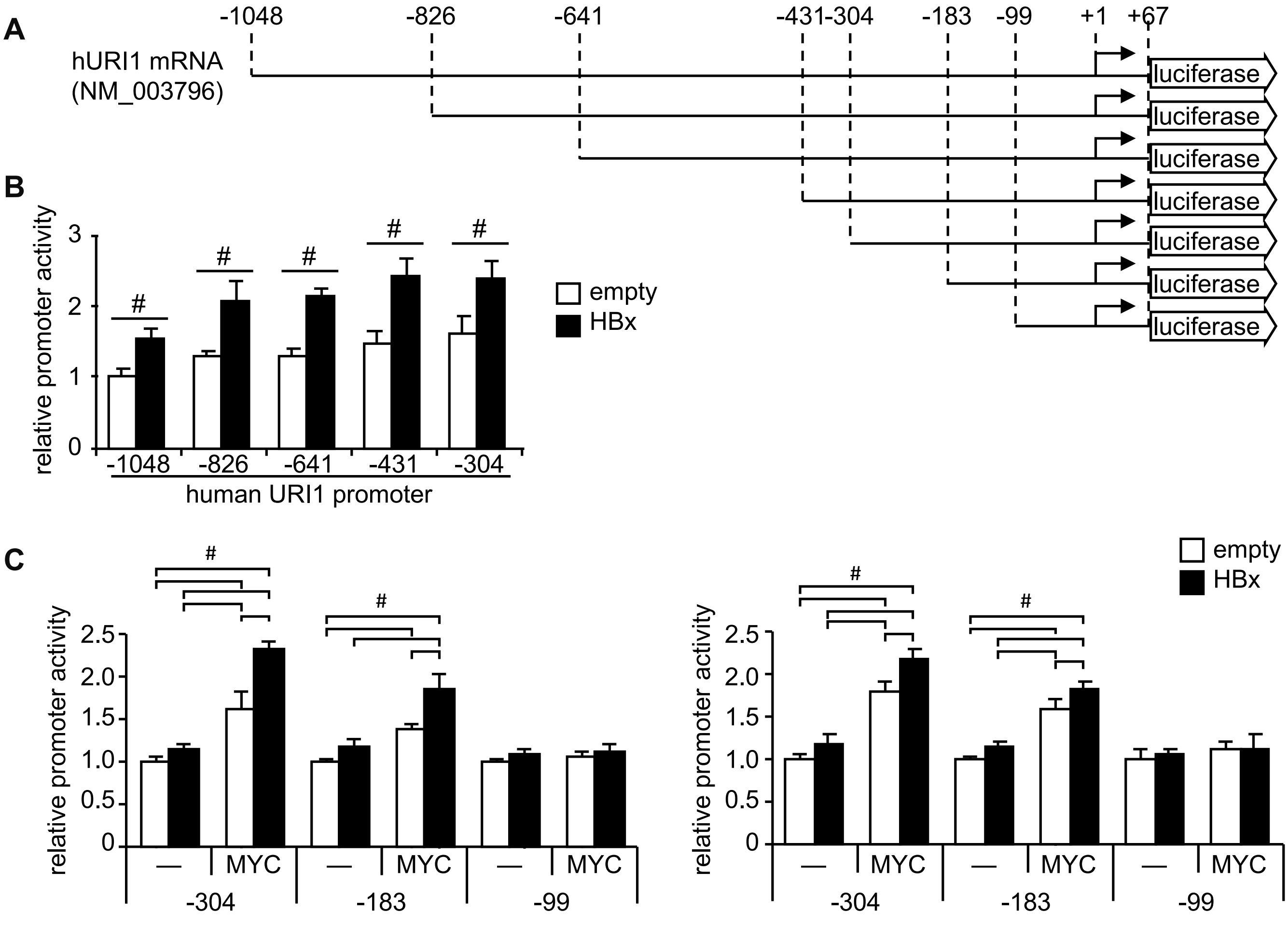

Supplementary Figure S1. Reporter assay for the *URI1* promoter. (**A**) Structures of reporter genes. (**B**) Promoter activation by HBx. Data are shown as mean ± SD (n = 3–4). #; $P < 0.05$ was determined by Student's *t*-test. (**C**) Promoter activation by HBx and c-MYC. Data are shown as mean ± SD (n = 3–4). #; $P < 0.05$ was determined by Tukey's test.

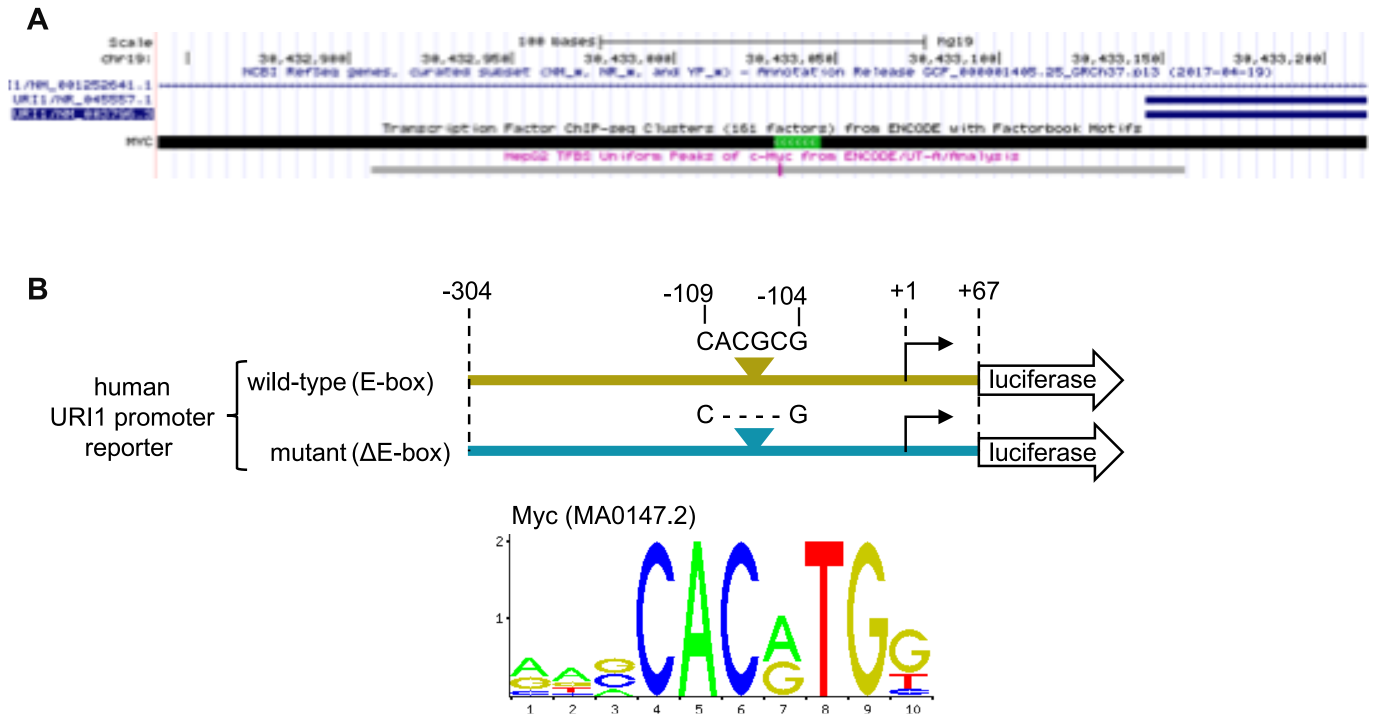

Supplementary Figure S2. c-MYC biding site in the *URI1* promoter. (**A**) c-MYC binding site demonstrated by the ENCODE project (GRCh37/hg19: chr19: 30,432,842-30,433,213). (**B**) Non-canonical E-box found in the *URI1* promoter using the JASPAR database.

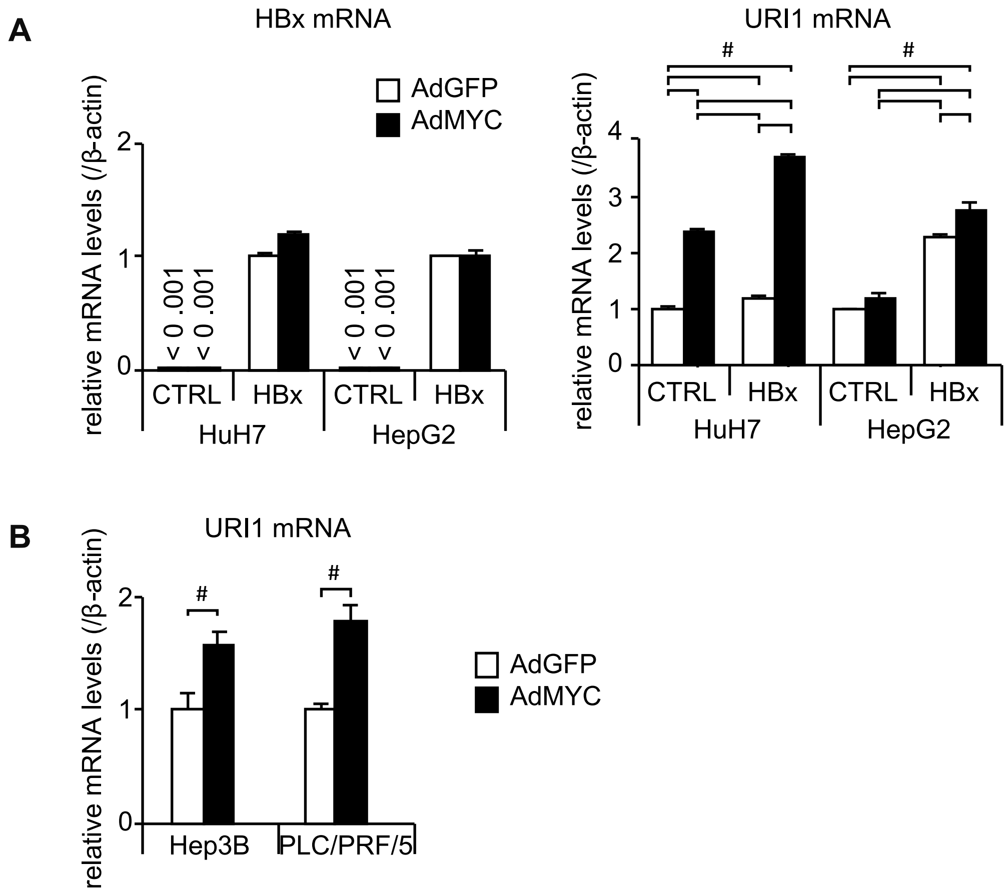

Supplementary Figure S3. Gene expression in HCC cell lines. (**A**) *HBx* and *URI1* mRNA expression in control (CTRL) or HBx-overexpressing HuH7 and HepG2 cells at 2 days post-transduction of AdMYC (MYC) or AdGFP (GFP). Data are shown as mean ± SD (n = 3). #; $P < 0.05$ was determined by Tukey's test. (**B**) *URI1* mRNA expression in Hep3B and PLC/PRF/5 cells at 2 days post-transduction of AdMYC (MYC) or AdGFP (GFP). Data are shown as mean ± SD (n = 3). #; $P < 0.05$ was determined by Tukey's test.
